# Visualization of Moyamoya Vessels Using Transcranial Color-Coded Duplex Sonography: A Case Report

**DOI:** 10.7759/cureus.91835

**Published:** 2025-09-08

**Authors:** Ryosuke Doijiri, Nobuharu Furuya

**Affiliations:** 1 Cerebrovascular Medicine, Akita Cerebrospinal and Cardiovascular Center, Akita, JPN; 2 Neurosurgery, Akita Cerebrospinal and Cardiovascular Center, Akita, JPN

**Keywords:** evaluation, magnetic resonance angiography, moyamoya disease, transcranial color-coded duplex sonography, visualization

## Abstract

Transcranial color-coded duplex sonography (TCCS) is a noninvasive modality that enables real-time evaluation of intracranial vasculature. In this report, we describe the bedside visualization of moyamoya vessels in a patient with established moyamoya disease using TCCS in combination with magnetic resonance angiography (MRA), highlighting the potential role of this approach in the detection of pathological collateral vessels. A 62-year-old woman with known moyamoya disease presented with acute impaired consciousness. MRA revealed bilateral terminal internal carotid artery (ICA) occlusion and prominent collateral vessel formation. TCCS demonstrated abnormal mosaic color flow in the middle cerebral artery (MCA) territory. The abnormal vascular signals observed with TCCS corresponded closely with the collateral network identified by MRA, supporting the concordance between these modalities. In this rare context of a postoperative bone defect created by prior right superficial temporal artery (STA) to MCA bypass surgery, TCCS enabled unusually detailed visualization of moyamoya vessels that corresponded closely with MRA findings. This case highlights the specific value of TCCS when a bone window is present, offering complementary diagnostic information that is not typically achievable in standard settings. This approach may serve as a valuable adjunct to conventional imaging in the evaluation and monitoring of moyamoya disease, particularly in clinical settings where bedside assessment is required.

## Introduction

Moyamoya disease is a progressive steno-occlusive cerebrovascular disorder that primarily involves the terminal portion of the internal carotid arteries (ICAs) and the proximal segments of the anterior and middle cerebral arteries (MCAs) [1]. As the disease progresses, an abnormal collateral vascular network develops at the base of the brain, producing the angiographic appearance described as “moyamoya vessels” [2]. Clinically, moyamoya disease typically presents with recurrent ischemic events in children and intracranial hemorrhage in adults, reflecting the fragility of the collateral network [3]. Digital subtraction angiography (DSA) remains the gold standard for the diagnosis of moyamoya disease, as it allows detailed visualization of steno-occlusive changes and the abnormal collateral network. However, magnetic resonance angiography (MRA) and computed tomography angiography (CTA) serve as valuable noninvasive alternatives for initial evaluation and follow-up, despite their lower spatial resolution compared with DSA [4].

Transcranial color-coded duplex sonography (TCCS) provides a real-time, noninvasive assessment of intracranial hemodynamics and has been recognized for its potential and limitations compared with conventional transcranial Doppler [5]. It has also been applied in selected cases of moyamoya disease [6,7]. However, there is a paucity of reports specifically addressing its use in patients with postoperative bone defects, where the absence of intact skull bone may create an enhanced acoustic window. To date, detailed descriptions of how such bone windows can facilitate more precise visualization of intracranial vessels compared with conventional TCCS through intact skull bone remain limited. Importantly, in the present case, the patient had previously undergone right superficial temporal artery (STA) to MCA bypass surgery, which resulted in a postoperative bone defect. This unique condition enabled unusually detailed visualization of moyamoya vessels by TCCS, a finding that would not typically be achievable in standard clinical settings.

## Case presentation

**Figure 1:**
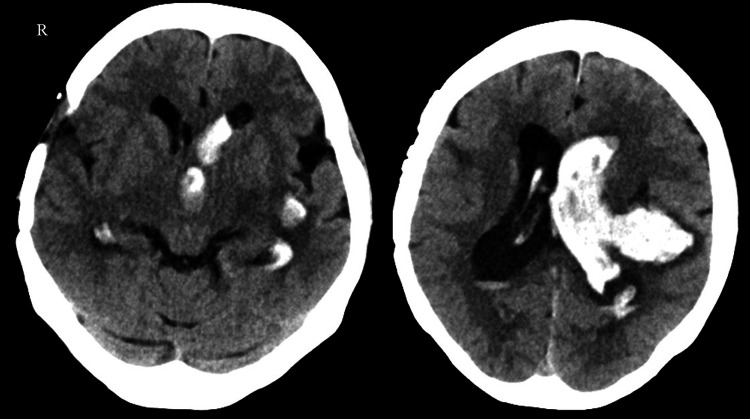
The patient's head CT on admission revealed a hemorrhage in the left lateral ventricle, leading to the diagnosis of cerebral hemorrhage associated with moyamoya disease. R indicates the right side.

A 62-year-old female patient with a medical history of hypertension and moyamoya disease had undergone right STA-MCA bypass surgery at an external institution. She was transported to the hospital by ambulance, having experienced a state of loss of consciousness. Upon admission, the patient exhibited a blood pressure reading of 136/77 mmHg, a Japan Coma Scale score of 100, right hemiplegia, and a National Institutes of Health Stroke Scale score of 18 [8]. A head CT scan revealed a left lateral ventricle hemorrhage, leading to a diagnosis of cerebral hemorrhage due to moyamoya disease (Figure 1). 

**Figure 2:**
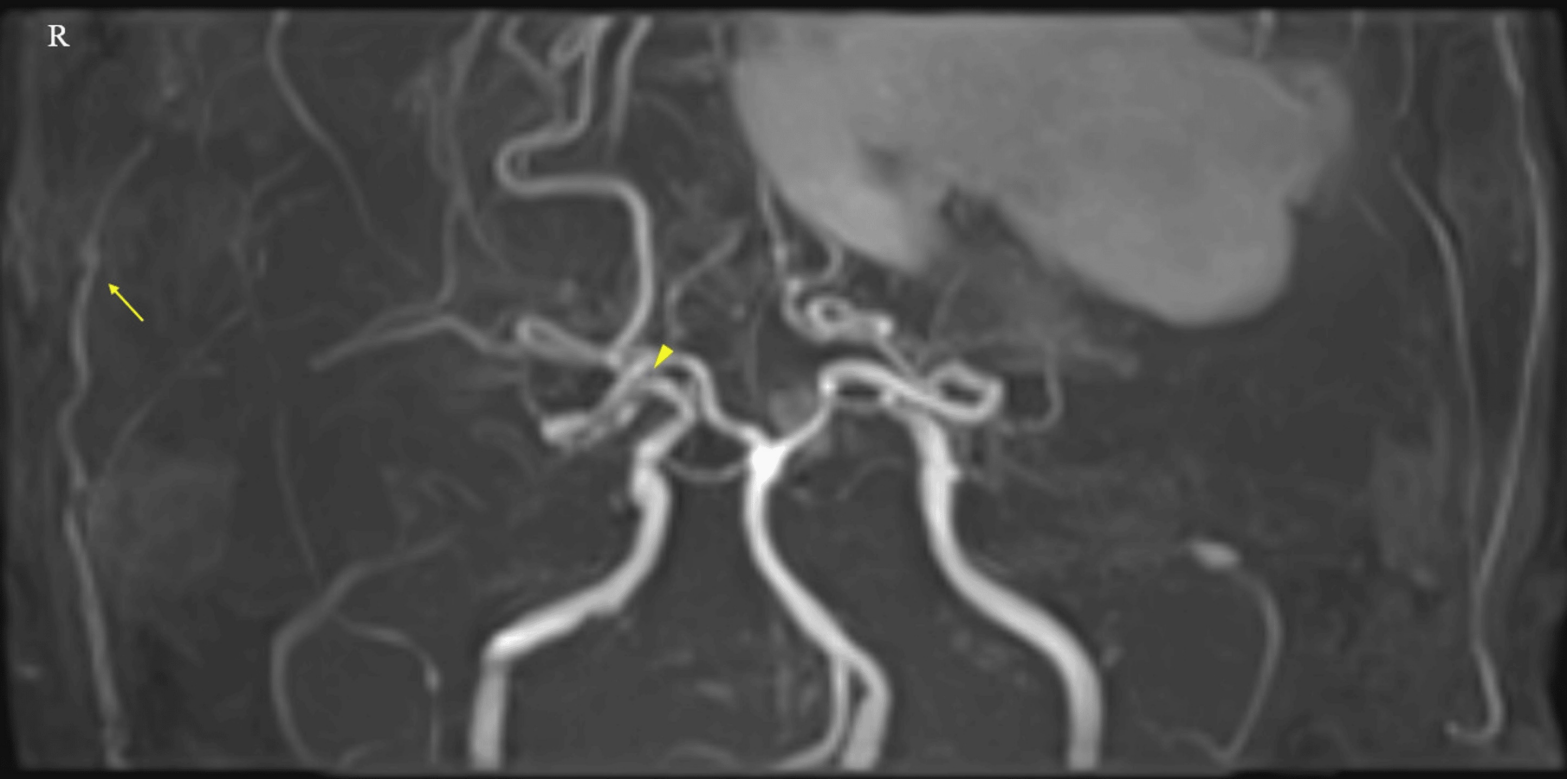
Magnetic resonance angiography (MRA) with maximum intensity projection (MIP) The MIP image demonstrates occlusion of the right internal carotid artery (ICA) at the C1 portion (arrowhead). The right superficial temporal artery is also visualized. On the contralateral side, the left ICA shows occlusion at the C1 portion, consistent with the diagnosis of moyamoya disease.

Subsequent to the patient's admission, MRA was performed, revealing the occlusion of the terminal portions of both ICAs and the proliferation of moyamoya vessels. Right STA was only slightly visible (Figure 2).

**Figure 3:**
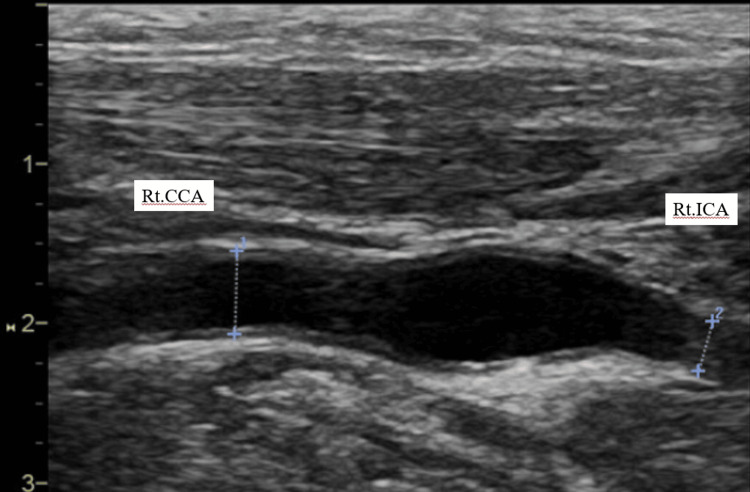
Carotid ultrasonography of the right (Rt.) common carotid artery (CCA), bifurcation, and internal carotid artery (ICA, long-axis view) Ultrasonography demonstrates an adventitia-to-adventitia diameter of 5.2 mm in the Rt. CC and 3.2 mm in the ICA, yielding a ratio of 0.61. This vascular morphology resembles the characteristic “champagne bottle neck” sign.

In the carotid ultrasound examination, the external diameter of the right common carotid artery (CCA) was 5.2 mm, and the diameter of the ICA was 3.2 mm, showing narrowing of the ICA and findings resembling the champagne bottle neck sign (Figure 3).

**Figure 4:**
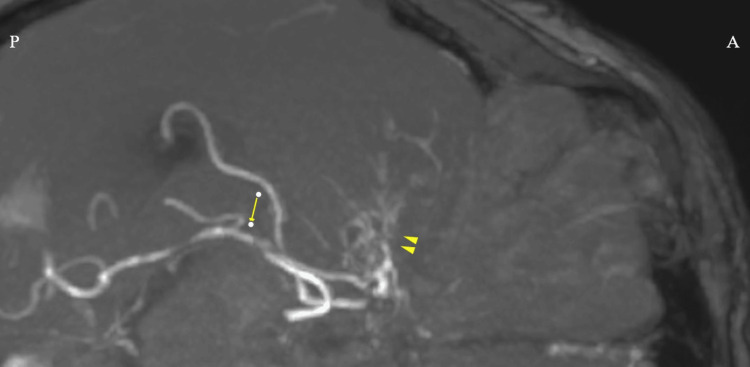
Time-of-flight (TOF) magnetic resonance angiography (MRA) of the head TOF MRA demonstrates proliferation of moyamoya vessels (arrowhead) in the region corresponding to the course of the right middle cerebral artery. The right posterior cerebral artery is also visualized (arrow). A indicates the anterior aspect, and P indicates the posterior aspect.

**Figure 5:**
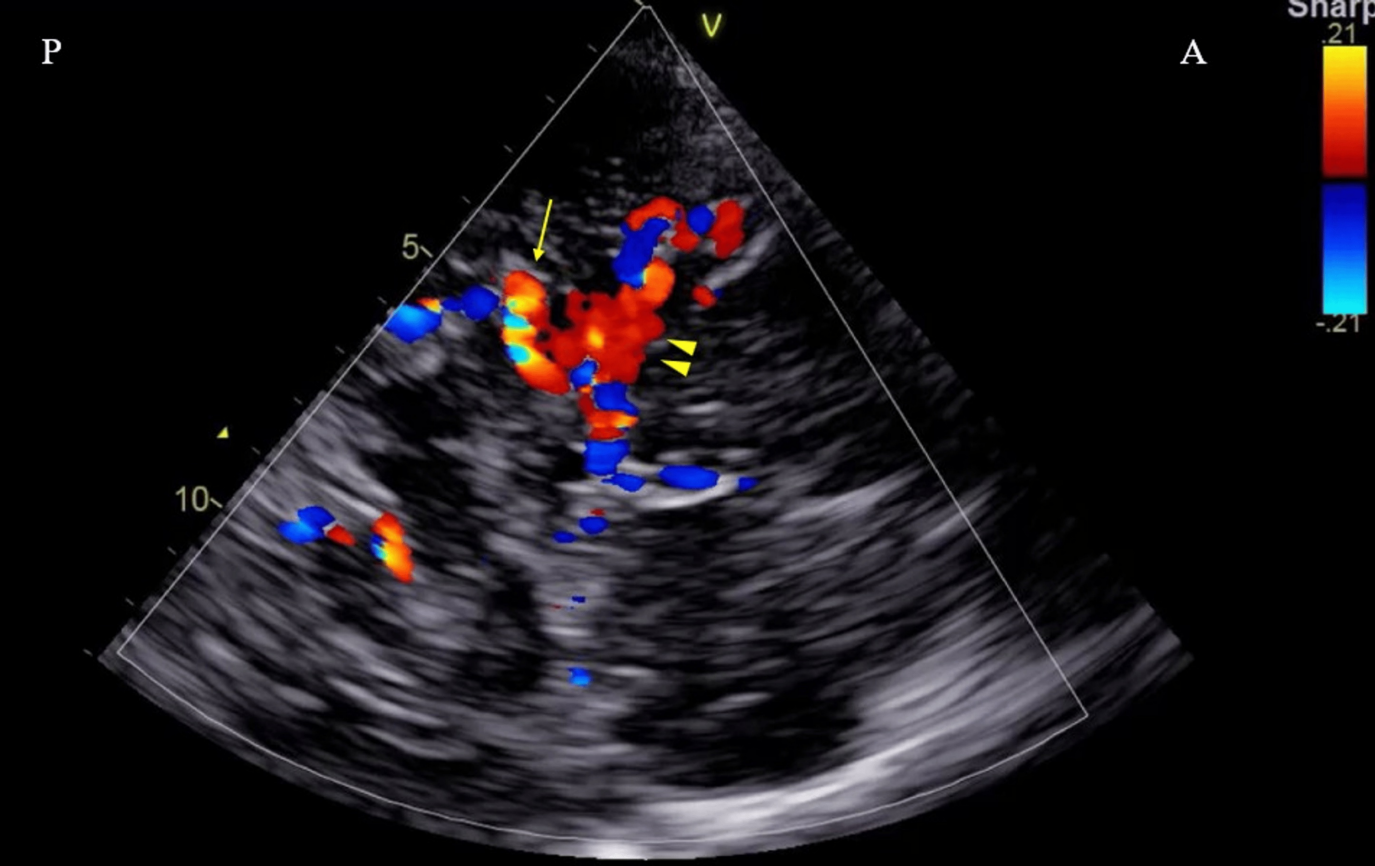
Transcranial color-coded duplex sonography (TCCS) through the right temporal window TCCS performed via the right temporal window through the postoperative bone defect created by prior superficial temporal artery to middle cerebral artery bypass surgery demonstrates moyamoya vessels (arrowhead) in the region corresponding to the abnormality identified on time-of-flight (TOF) magnetic resonance angiography. In addition, the right posterior cerebral artery is visualized (arrow). A represents the anterior aspect, and P represents the posterior aspect.

TCCS revealed abnormal color flow signals consistent with moyamoya vessels in the region corresponding to the course of the MCA. These signals demonstrated a mosaic pattern suggestive of slow and multidirectional collateral circulation. The observed abnormal flow corresponded closely with the vascular network identified on time-of-flight (TOF) MRA of the head (Figures 4, 5).

These findings suggest that the detection of moyamoya vessels by TCCS may provide additional diagnostic information when used in conjunction with conventional neuroimaging modalities such as MRA.

## Discussion

This case underscores the potential utility of TCCS with color flow imaging as a bedside modality for the evaluation of moyamoya disease. A unique aspect of the present case is that the patient had undergone STA-MCA bypass surgery, which created a postoperative bone defect. This condition substantially enhanced the acoustic penetration and allowed unusually detailed visualization of moyamoya collaterals by TCCS. In this report, contralateral TCCS was not performed, as the clinical examination was focused on the symptomatic side with a prior bypass procedure. This represents a limitation of our study, since direct comparison with the intact side could not be made. Nonetheless, the absence of contralateral data should be interpreted in the context of the present case, in which the postoperative bone defect uniquely allowed unusually detailed visualization of moyamoya vessels by TCCS, an observation that would not typically be achievable through an intact skull. In typical cases without such a bone window, the intact skull markedly limits ultrasound transmission, making comparable visualization difficult. Therefore, this report demonstrates how a postoperative bone window can provide an exceptional opportunity to evaluate moyamoya vessels using TCCS. The abnormal collateral vessels visualized in the MCA territory corresponded closely with the pathological “moyamoya vessels” identified on MRA, thereby demonstrating concordance between these imaging approaches. An important consideration in the sonographic assessment of intracranial vasculature is the distinction between true pathological flow signals and imaging artifacts. To minimize false-positive findings, careful adjustment of insonation angle, color gain, and wall filter settings is essential [9].

Although TCCS is widely recognized for its real-time assessment of intracranial hemodynamics, its diagnostic utility in moyamoya disease has been variably reported. Previous studies have demonstrated that TCCS or power Doppler can detect hemodynamic alterations and, in some cases, moyamoya-like collaterals [6], and Braun et al. reported a case of moyamoya syndrome in which ultrasound identified obliterated intracranial arteries and collateral circulation consistent with angiographic findings [7]. In addition, emerging techniques such as microvascular flow imaging (MVFI) have shown promise in improving the sensitivity for detecting low-velocity and multidirectional flow, thereby providing more detailed visualization of pathological collateral vessels compared with conventional Doppler modalities [10]. Beyond the MCA territory, other vascular structures, including the intracranial ICA and posterior cerebral artery, may provide complementary diagnostic information in moyamoya disease [2].

Nevertheless, several limitations should be acknowledged. The present report describes a single case, and its findings may not be generalizable to all patients with moyamoya disease. In addition, the diagnostic accuracy of TCCS is influenced by bone window quality and operator expertise, which can limit reproducibility. Finally, while TCCS offers a valuable adjunctive approach, digital subtraction angiography remains the diagnostic gold standard, with MRA and CTA serving as reliable noninvasive alternatives for screening and longitudinal follow-up [4].

In conclusion, this case demonstrates that a postoperative bone defect can provide a unique acoustic window that enables unusually detailed bedside visualization of moyamoya vessels by TCCS. While TCCS in general may offer complementary diagnostic information, this report emphasizes its specific utility in the rare context of a surgically created bone window.

## Conclusions

This case demonstrates that in the presence of a postoperative bone defect, TCCS with color flow imaging can provide unusually detailed bedside visualization of moyamoya vessels. While DSA remains the diagnostic gold standard and MRA serves as a valuable noninvasive alternative, our findings emphasize the specific clinical value of TCCS in the rare context of a surgically created bone window. The integration of TCCS into clinical practice could enhance real-time hemodynamic assessment and serve as a practical adjunct for the evaluation and monitoring of moyamoya disease. In particular, TCCS may be of clinical value in patients with postoperative or other bone defects, where enhanced acoustic windows can allow more detailed intracranial vascular assessment.
